# Advances in Alternative Measures in Plant Protection

**DOI:** 10.3390/plants12040805

**Published:** 2023-02-10

**Authors:** Dragana Šunjka, Špela Mechora

**Affiliations:** 1 Faculty of Agriculture, University of Novi Sad, Trg Dositeja Obradovića 8, 21000 Novi Sad, Serbia; 2 Agency for Radwaste Management, Litostrojska 58A, 1000 Ljubljana, Slovenia

Food production, along with the constant demand for higher yields, is an imperative of contemporary agricultural production. In order to accomplish this goal, it is necessary to control pests, weeds, and the causative agents of plant diseases, which implies the application of plant protection products. However, the intensive and/or unsuitable application of synthetic pesticides has led to a series of consequences for agricultural production itself and for the environment, non-target organisms, and for human health as well. The presence of resistant populations, side effects on pollinators and other beneficial organisms, and pesticide residues in food, water, and soil are just some of the consequences. The fact that in recent years the number of active substances approved for use in agricultural production is rapidly decreasing, all of the above requires continuous and comprehensive studies of the response of the living organism (beneficial and harmful effects) to various new agrochemicals and to products of biotechnology, followed by the mandatory risk assessment of the effect on humans and animals as well as the environment. 

In order to minimize the use of chemical pesticides, the Special Issue “Advances in Alternative Measures in Plant Protection” intends to offer an insight into the alternative measures of plant protection in contemporary agriculture. Special Issue contains twenty-two scientific articles: sixteen original research articles and six original reviews. With these research and reviews, authors contribute to the development of new, biological ways of controlling pests in agricultural production.

As an alternative to synthetic pesticides for the control of plant diseases, especially for the management of plant pathogenic fungi, bioactive metabolites derived from algae/plant extracts, essential oils, and other materials can be used. The extract of seaweed *Gracilariopsis persica* has drawn attention for its biologically active compounds which show antifungal activities against *Botrytis cinerea*, *Aspergillus niger*, *Penicillium expansum*, and *Pyricularia oryzae* on the mycelial growth. The antifungal activity is most likely associated with its phenolic compounds, such as rosmarinic, oleic, and palmitic acid and quercetin, whose antifungal activity has already been reported [1]. The antifungal effect of phenolic compounds is potentially reflected in the reduction in the wood mass loss and restriction of wood biodegradation caused by *Ganoderma boninense* in inoculated oil palm woodblocks [2]. The hydromethanolic extract of *Rubia tinctorum* root also shows antifungal activity, alone or in combination with chitosan oligomers or with stevioside, against three *Botryosphaeriaceae* taxa by the inhibition of their mycelial growth. Considering the complexity of the extracts' chemical composition, the activity most likely should be ascribed to the combination of a group of them [3]. In addition to the antifungal effects, phenolic compounds play a notable role in the resistance of plants to diseases. The phenolic compound content usually increases after powdery mildew disease occurs, which leads to the conclusion that hybrids/cultivars with high phenolic contents should be recommended for the development of new superior cultivars as a source of resistance to fungal grape diseases [4]. 

An important part of sustainable pest management is natural enemies. Root fungal endophytes isolated from solanaceous plant species expressed activity in the control of a late blight of potato incited by *Phytophthora infestans* (Mont.) de Bary [5]. When it comes to the natural regulation of pests, it is particularly important to provide suitable conditions for beneficial organisms. Field margin plant species are considered to be food and shelter for specific pests; therefore, it is crucial to study the biology of the host plants and how they interact with pests [6]. 

For the management of plant pathogens, as an environmentally friendly method of plant disease control, microorganisms can be used usually because of their capability of counteracting the growth. Species belonging to the bacterial genus *Pseudomonas* have a high potential in the control of plant pathogens. They can be used as an alcoholic extract derived from bacterial isolate, which manifests exceptional activity against some of the most common pre- and post-harvest fungal diseases [7]. 

Indoor agricultural production is particularly challenging due to conditions affordable for pests. As an alternative to chemical products, the control of causal agents of the major tomato diseases in greenhouse production was undertaken by *Bacillus* spp. or *B. subtilis* and the foliar application of *Reynoutria sachalinensis*, *Melaleuca alternifolia*, harpin αβ proteins, and bee honey. The use of these biorational products in the control of these diseases in greenhouse production has the potential to be incorporated into an integrated program for the management of the examined diseases in tomatoes [8]. Moreover, in the same conditions, the control of *Sclerotium rolfsii*, a destructive disease for many plants, including tomatoes, could be successfully undertaken using some antiseptic and disinfectant agents [9].

Plant extracts can indicate an herbicide effect as well. *Thymus vulgaris* hydrolate showed an inhibitory effect on the weed species, including *Amaranthus retroflexus* (L.), *Chenopodium album* (L.), *Portulaca oleracea* (L.), *Echinochloa crus-galli* (L.) P. Beauv., *Sorghum halepense* (L.) Pers., and *Solanum nigrum* L., through the inhibition of the seed germination and seedling growth. *T. vulgaris* hydrolate had the least negative phytotoxic effect on the germination of soybean, sunflower, and maize [10]. 

Understanding the plant–plant interaction is also one of the Advances in Alternative Measures in Plant Protection. The phytotoxic substances released by *Ambrosia trifida* L. have an allelopathic influence on the oxidative stress parameters and phenolic compounds in maize, soybean, and sunflower. The sunflower was the most sensitive crop to *A. trifida* allelochemicals, maize showed a mild sensitivity, while the soybean did not demonstrate sensitivity [11]. The phytotoxic effect could perhaps be reduced using some biostimulants. L-α-amino acid-based biostimulants show a protective effect against the stress caused by the application of the imazamox herbicide in sunflower plants, followed by the protection of the photosynthetic activity and reduction in the oxidative stress in the plant [12]. Furthermore, even some strains of entomopathogenic fungi can act as biostimulants for some crops by their ability to infect insect pests and to promote plant growth. For example, the isolate of *Metarhizium robertsii* (2693) causes the death of 73% *Tenebrio molitor* larvae, but also significantly increases the maize root length [13]. 

Aside from plants, microorganisms, insects, and nematodes, micronutrients have to be considered as a source of bioactive compounds. Recently, some micronutrients are recognized as an alternative to synthetic fungicides. Due to the influence of Selenium on the inhibition of the development of a *Fusarium proliferatum*, the addition of low concentrations of Se could improve conventional fungicides and decrease their side effects [14]. One of the most significant economic and ecologic forest tree species in Europe, the sessile oak, is highly threatened by the powdery mildew caused by *Erysiphe alphitoides* (Griffon and Maubl.). Evaluating the influence of the irrigation levels (overhead sprinklers) on the damage caused by powdery mildew to *Quercus petraea* in a nursery setting, it was showed that controlling the irrigation rate can become an effective component of integrated protection strategies against this pathogen [15]. The negative impact on the citrus production caused by pests *Tetranychus urticae* Koch 1836 could be successfully controlled by eco-friendly approaches, such as black soap and detergents, without having a negative impact on predators [16]. 

Furthermore, the authors review the latest knowledge regarding the importance of plant-parasitic nematodes in agricultural production [17] and genetically based plant resistance underlying plant–nematode interactions [18] as a safe alternative to chemical nematocides and describe the potential of using *Photorhabdus* spp. as biocontrol agents against a broad range of insect pests [19]. As an effective source of biofungicide, the authors provide recent knowledge of actinomycetes and their potential as biocontrol agents of phytopathogenic fungi [20].

Finally, the utility of biocontrol agents composed of microorganisms, plant-based compounds, as well RNAi-based technology has to be emphasized [21], not only in an organic agriculture, where they represent the exclusive measure of plant protection, but also in integrated and conventional agriculture. However, the main task in the field of plant protection, and the most challenging at the same time, is still finding alternative sources of compounds with pesticide activity and the improvement of their formulation [22]. 

As Guest Editors, we are grateful to all the authors for choosing this Special Issue to publish their research. We hope that together we have contributed to the understanding of the importance of alternative measures in plant protection and opened up new possibilities for the improvement of this field. 
